# The influence of Bouba- and Kiki-like shape on perceived taste of chocolate pieces

**DOI:** 10.3389/fpsyg.2023.1170674

**Published:** 2023-06-15

**Authors:** Kazuhiro Ogata, Reo Gakumi, Atsushi Hashimoto, Yoshitaka Ushiku, Shigeo Yoshida

**Affiliations:** ^1^Department of Design, Kyoto Institute of Technology, Kyoto, Japan; ^2^OMRON SINIC X Corporation, Tokyo, Japan; ^3^Graduate School of Informatics, Kyoto University, Kyoto, Japan

**Keywords:** crossmodal correspondences, Bouba-Kiki effect, chocolate, shape-taste correspondences, 3D food printer

## Abstract

In this paper, we present the findings of a study investigating the impact of shape on the taste perception of chocolate. Previous research has explored the influence of various sensory information on taste perception, but there has been little focus on the effect of food shape being eaten on taste perception. To explore this, we focused on the Bouba-Kiki effect, illustrating an interaction between shape and several modalities, and investigated the effect of Bouba- and Kiki-shaped (rounded and angular) foods eaten on taste perception. We utilized a 3D food printer to produce four different shapes of chocolate pieces based on the Bouba-Kiki. Participants tasted each piece and completed a chocolate flavor questionnaire. With Bayesian analysis, we determined that the Bouba-shaped chocolate pieces were perceived as sweeter than the Kiki-shaped ones, supporting earlier studies on crossmodal correspondences between shape and taste perception. However, there were no significant differences in ratings of other tastes, such as sourness and bitterness. Our research indicates that shape can affect taste perception during consumption and suggests that 3D food printers offer an opportunity to design specific shapes that influence taste experiences.

## 1. Introduction

The experience of food results from the integration of multisensory information. Previous studies have shown that the visual and tactile characteristics of food [e.g., color: Spence et al. (2015), Velasco et al. (2016a), shape: Gal et al. (2007), Spence and Gallace (2011), Spence and Deroy (2014), and its texture: Slocombe et al. (2016)] affect taste perception. Extrinsic or environmental characteristics when eating also influence taste perception. For example, studies of taste perception and related experiences have demonstrated that the taste of food influenced by the characteristics of the cutlery (e.g., weight, size, shape, and color: Harrar and Spence, 2013; Michel et al., 2015b), how food is arranged on the plates (Michel et al., 2015a), environmental sound (Mesz et al., 2011; Bronner et al., 2012), atmosphere, and room lighting (Velasco et al., 2013; Spence et al., 2014; Nygård and Lie, 2020), product packages and labels (Velasco et al., 2016b), and congruency between content and glassware (Wan et al., 2015; Van Doorn et al., 2017). Such non-arbitrary associations among different modalities are called “crossmodal correspondences”, and various associations among modalities have been demonstrated for senses other than taste (Spence, 2011).

The Bouba-kiki effect is a well-known example of crossmodal correspondences that illustrate an interaction between shape and speech sound. Bouba-Kiki was derived from an earlier study (Köhler, 1929) that presented many people with similar associations between two non-sense words or pseudowords (Maluma/Taketa) and two types of geometric shapes (rounded/angular). Subsequently, Ramachandran and Hubbard (2001) mentioned that 95% of their participants responded to the same associations between the two types of rounded or angular shapes and speech sounds. Studies have extended the Bouba-Kiki effect to reveal correspondences between foods/flavors and words (Gallace et al., 2011), flavors of liquids (Deroy and Valentin, 2011), and between rounded/angular shapes and cheese taste (Spence et al., 2013). Moreover, some other studies have investigated the Bouba-Kiki effect in terms of different transparent window shapes in product packaging (Simmonds et al., 2019) and the effect of different bowl shapes on the expected taste for a dessert (Chen et al., 2018). The results showed correspondences between sweetness and rounded shapes and bitterness/sourness and angular shapes. Several reports have also demonstrated similar results (Ngo and Spence, 2011; Ngo et al., 2011; Wang et al., 2017). In addition, Salgado-Montejo et al. (2015) conducted an experiment on the Bouba-Kiki effect using two additional properties of the shapes and shape symmetry (Makin et al., 2012), and number of elements (Jacobsen et al., 2006; Palmer et al., 2013).

Many studies have demonstrated a relationship between the appearance and shape of food and its taste, including the Bouba-Kiki, but it remains unclear how taste changes when such shaped foods are consumed. For example, Lenfant et al. (2013) demonstrated that different shapes (e.g., rectangle, triangle, round) of dark chocolate pieces affect flavor and texture. In contrast, Baptista et al. (2022) revealed that differences in shape have an impact on expected/perceived creaminess but not on sweetness, bitterness, and likability through a large-scale experiment. Wang et al. (2017) suggested there are no differences in the taste ratings of different shaped (rounded and angular) chocolate pieces when consumed, although ratings of expected taste before eating did differ by shape. In contrast, a recent study reported that the 3D Bouba-shaped edible samples were perceived as sweeter than the Kiki-shaped samples (Cornelio et al., 2022). However, the edible sample was prepared and designed only for the experiments (the ingredients were water, pork gelatin, agar, and sugar); unlike foods normally consumed, the taste was relatively neutral. Hence, it is unclear whether the Bouba-Kiki effect applies to the taste perception of foods usually consumed.

**Figure 1:**
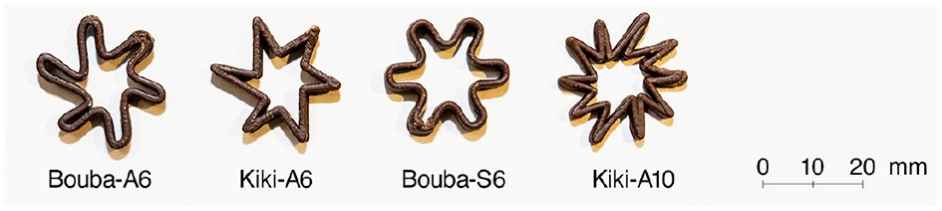
Chocolate pieces used in the study. The alphabetical letter following the hyphen means symmetrical (S) and asymmetrical (A), and the number means the number of elements (6 or 10 in this study).

In this paper, we investigated the effects of Bouba-Kiki-shaped edible samples of a commonly consumed food item (i.e., chocolate, Figure 1) on taste perception. Chocolate was chosen as a material for the edible samples because chocolate has some inherent flavors and aromas, and has been used in previous taste perception studies, as described above. Chocolate is a common material used for 3D food printing (Hao et al., 2010; Lanaro et al., 2019; Mantihal et al., 2019; Souto et al., 2022). Chocolate as a 3D food printer material enables easy to control of shape and permits the production of a large number of edible samples for experiment.

As discussed above, several studies of crossmodal correspondences have demonstrated that Bouba (rounded) shapes are associated with sweetness and Kiki (angular) shapes are associated with sourness/bitterness. Thus, we hypothesized that Bouba-shaped chocolate pieces would be rated as sweeter than Kiki-shaped pieces (*H*_1_), whereas ratings of sourness and bitterness would be greater for Kiki- than for Bouba-shaped pieces (*H*_2_:sourness/*H*_3_:bitterness). In addition, based on the premise that rounder, symmetrical, and those shapes with fewer elements are more likely to be judged as sweeter and angular, asymmetrical, and those shapes with a greater number of elements are more likely to be judged as more sour (Salgado-Montejo et al., 2015), we assumed that the symmetrical Bouba-shaped chocolate piece was more likely to be perceived as sweeter than the asymmetrical Bouba-shaped sample (*H*_4_) and the Kiki-shaped sample with a greater number of elements would be perceived as more sour than that with fewer elements (*H*_5_). Similarly, the effect of shape (an asymmetric shape with more elements) on bitterness was also investigated (*H*_6_).

## 2. Materials and methods

### 2.1. Taste stimuli

#### 2.1.1. Stimulus design

We designed four Bouba/Kiki-shaped stimuli for this study (Figure 1). These shapes are derived from geometric forms, as shown in Köhler (1929). Each shape we designed was named according to its Bouba/Kiki form (i.e., rounded/angular), symmetry/asymmetry, and number of elements. For example, Bouba-S6 denotes a symmetric Bouba-shaped chocolate piece with six elements, whereas Kiki-A10 denotes an asymmetric Kiki-shaped chocolate piece with 10 elements.

First, two shapes with rounded or angular tips of the same basic shape (Bouba-A6 and Kiki-A6 in Figure 1) were chosen as a standard edible samples. This was consistent with our basic hypothesis, namely that a Bouba-shaped edible sample would be perceived as sweeter than an equivalent Kiki-shaped sample (conversely, a Kiki-shaped sample was hypothesized to be perceived as more sourer/bitter than the equivalent Bouba-shaped one). In addition, stimuli with modified symmetry and number of elements were designed (Bouba-S6 and Kiki-A10) because prior work demonstrated differences in taste expectation by changing the symmetry/asymmetry and number of elements of Bouba-Kiki-shaped stimuli (Salgado-Montejo et al., 2015). Therefore, we focused on these parameters, which had a particular influence on sweetness and sourness/bitterness in prior work, and designed symmetric Bouba-shaped stimuli with few elements (6 in our study), and asymmetric Kiki-shaped stimuli with a large number of elements (10 in our study).

Prior studies found no differences in participants' reports of taste differences following actual consumption of round and angular chocolate pieces (Wang et al., 2017). We assumed that the amount of chocolate eaten in this prior study was too large, which caused the actual taste to dominate any effect of perceived shape on taste. Therefore, we designed a ring-shaped stimulus that was not filled with chocolate at its center to avoid the need to consume excessive chocolate taste/flavor, while maintaining perceived differences in shape.

#### 2.1.2. 3D modeling and printing

Here, we briefly explain the conditions for 3D modeling and chocolate printing.

**Figure 2:**
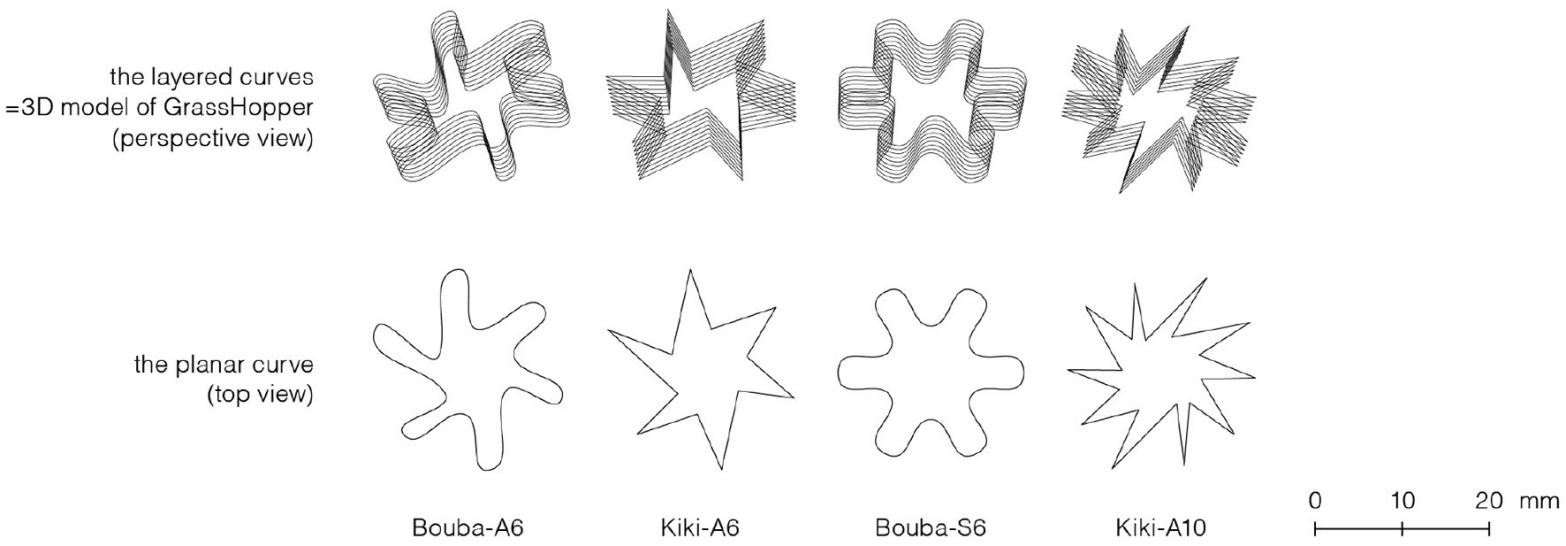
3D model and its planar curve used to print the chocolate pieces.

We used 3D CAD software (Rhinoceros^1^) and its plug-in (Grasshopper^2^) to design the stimuli. The edible samples were 24 mm in width and depth, and 3–4 mm in height (Figure 2). This volume was determined based on the results of a pilot study. Participants found it easy to place the samples in their mouth in one bite. The height was designed to be greater than the minimum discriminable distance between two points on the tongue, namely 1.650 ± 0.433 mm at the tip of the tongue and 2.650 ± 0.856 mm and 1.675 ± 0.269 mm at the sides of the anterior margins of each side of the tongue (Maeyama and Plattig, 1989); thus differences in texture could be perceived. The minimum two-point discrimination distance was also used to determine the distance between the tips of the shape, and the number of elements. Additionally, to equalize the weight of each sample, the height of each sample was controlled by varying the number of layers from 7 to 10. The symmetry/asymmetry and number of elements of the Bouba-Kiki shapes were partly determined with reference to a previous study (Salgado-Montejo et al., 2015).

To obtain precise and flexible control over the shape, mass, and volume when producing ring-shaped edible samples, a 3D food printer (Foodbot^3^) was used. This printer is designed for printing edible ingredients using a temperature-controllable food-grade syringe. The nozzle can also be changed to permit the printing of food objects with subtle textures and smooth surfaces. We used the nozzle of diameter of 0.6 mm in this study to make the edge of the Kiki-shaped samples precisely angular. The printer can directly read G-code files, which is a geometric code that controls printer direction, speed, and other relevant parameters (Derossi et al., 2019). In many cases, G-code is generated using slicing software. However, 3D objects can also be created and G-code generated using 3D CAD software and plug-ins. Printing conditions were determined based on previous studies that fabricated chocolate using 3D printers (Mantihal et al., 2017; Lanaro et al., 2019). Our printing conditions were as follows: print speed from 12.0 to 15.0 mm/s, extrusion multiplier of 0.8%, and layer pitch of 0.42 mm. Room temperature during printing maintained below 25.0°C to prevent the chocolate from melting.

#### 2.1.3. Ingredients

The chocolate used as the edible sample was a commercially available 74% cacao couverture chocolate (ingredients: organic cacao mass, organic cane sugar, organic cacao butter, organic cacao powder, organic vanilla powder). This chocolate was used because lower-purity cacao complicates the tempering and printing process (Souto et al., 2022). That is, it is necessary to perform tempering to achieve crystallization of a stable form of chocolate that can be precisely printed. First, chocolate was melted at approximately 45°C, after which the temperature was reduced to 34°C. The chocolate was mixed with 1% (by weight of chocolate) cocoa butter powder as a seed crystal to produce stable form of chocolate (Lanaro et al., 2019). The temperature was maintained at 33°C at the end of this process. Mixing cocoa butter simplifies the tempering process and helps promote the growth of Form V crystal nuclei. The tempered chocolate was poured into a plastic syringe and printed using the 3D food printer.

### 2.2. Experimental setup and design

The experiment was conducted in a quiet room with closed blinds. The air conditioning was set at 23°C, and a fan was running quietly in the background. The participants were seated in the room and tasted one edible sample at a time and rated the samples using a questionnaire presented on a tablet. We collected demographic information (age and sex) and taste ratings for each sample. Participants rated the following questions in a visual analogue scale (VAS) ranging from 0 to 100:

1. How much sweetness did you perceive in the chocolate?
2. How much sourness did you perceive in the chocolate?
3. How much bitterness did you perceive in the chocolate?

The experimenter placed the edible samples individually on a disposable plastic spoon (white, 160 mm long, 38 mm wide, for hygienic reasons) on a paper plate in front of the participant. The edible samples were presented at room temperature. The experiment was conducted using a within-participants design. Each participant consumed four samples with different shapes. The order in which the participants consumed the samples was predetermined by Latin square design, resulting in 24 possible sequences (factorial of 4 = 24).

### 2.3. Participants

Twenty-four Japanese participants (12 male, 12 female; age range 22–47 years, average age 33 years) without chocolate or dairy product allergies were recruited for this study. They were requested not to consume spicy food 24 h prior to the experiment, alcohol 6 h prior, or any food or drink (except water) 1 h prior (Obrist et al., 2014). Ethical approval was obtained from the Research Ethics and Review Committee of our company and written informed consent was obtained from all participants.

### 2.4. Procedure

**Figure 3:**
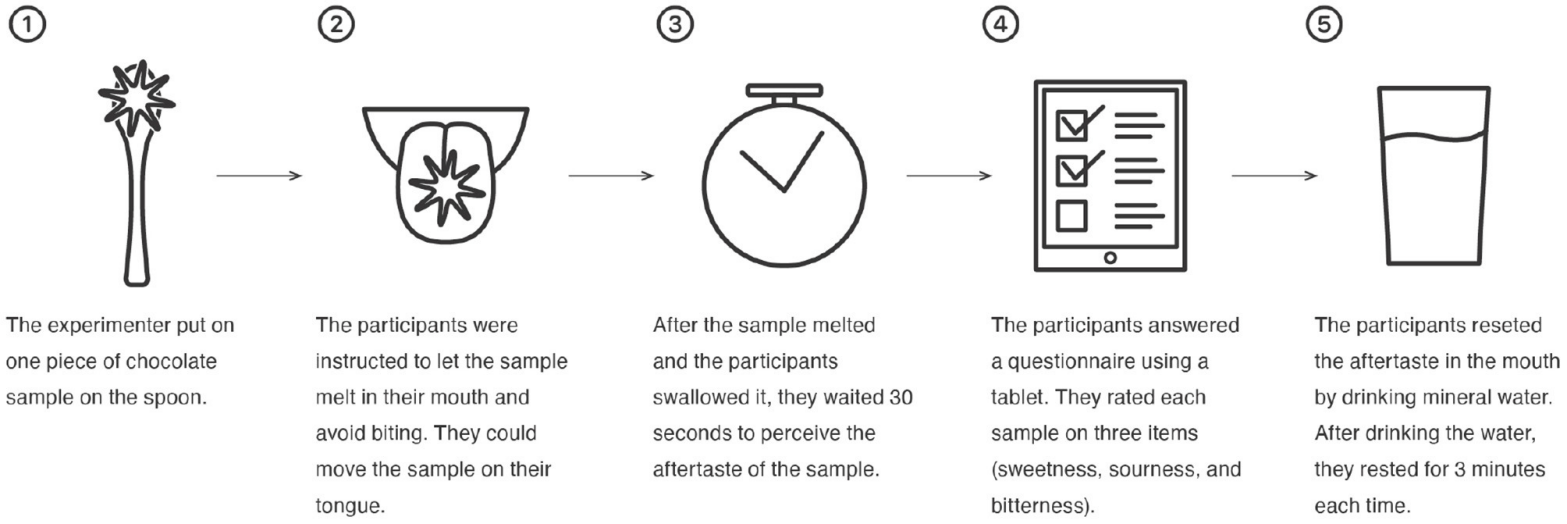
The experimental procedure followed by the participants. Step 1 to 5 were repeated four times.

The procedure of the experiment was as follows (Figure 3):

1. The experimenter put one piece chocolate sample on the spoon.
2. The participant was instructed to bring the sample to their mouth with the spoon and put it on their tongues. They were instructed to let the sample melt in their mouth and avoid biting. They were allowed to move the sample into their mouths, and the movement of their tongue was not restricted.
3. After the sample was melted and the participant swallowed it, they were instructed to wait 30 s to perceive the aftertaste of the sample.
4. The participant was then asked to complete a questionnaire that was presented on a tablet. They rated each sample in terms of three characteristics (sweetness, sourness, and bitterness).
5. The participant was asked to clear the aftertaste from their mouth by drinking room-temperature mineral water. After drinking the water, they remained in their seats and rested for 3 min each time to avoid any carry-over effect. In this study, we used room-temperature water, which is effective for cleaning the taste of astringent foods (Lee and Vickers, 2010).

The experiment consisted of repeating steps 1–5 four times and was completed in approximately 30 min. The study was conducted in Japanese, and the questionnaires used in the study were written in Japanese.

### 2.5. Analysis

We performed a statistical analysis^4^ of the experimental results using Bayesian analysis with Markov chain Monte Carlo methods (MCMC). We employed Bayesian analysis as it facilitates a natural interpretation of the probability that the research hypothesis holds true, and it directly provides the probabilistic range of the parameters, enabling flexible hypothesis evaluation.

We used Python3 (v3.8) for data preprocessing and Bayesian analysis via PyMC (v4.1),^5^ which is a Python package for the Bayesian statistical modeling of advanced MCMC. Five chains of 5,000 samples were generated using PyMC. The burn-in period was set to 1,000, 20,000 random numbers obtained using the No-U-Turn Sampler (NUTS) method were used to approximate the posterior and predictive distributions. The convergence in sampling was diagnosed using the Gelman-Rubin statistic, $|\hat{R}-1|<0.01$ for all parameters, and visually confirming indicate that the each Markov chain was converging to a stationary state properly.

It was assumed that each edible sample has its estimated value of population mean and population standard deviation denoted as μ_*i*_, σ_*i*_, and each of them follows a uniform distribution as a prior distribution.


$$\begin{array}{cc} \mu_{i} & \sim \text{Uniform}\left(0,100\right)\\ \sigma_{i} & \sim \text{Uniform}\left(0,50\right) \end{array}$$

The calculation of the multivariate normal log-likelihood that we used as the likelihood in our analysis requires a vector of means and a variance-covariance matrix. Since the variance-covariance matrix can be generated from the correlation matrix and the variance of each variable, we used the LKJ (Lewandowski-Kurowicka-Joe) correlation distribution (Lewandowski et al., 2009) as a prior distribution for the correlation matrix.

$u_{\mu_{i}-\mu_{j}}^{\left(t\right)}>0$ was set to compare the population means of the two edible samples. The probability that the hypothesis is true, *p*(μ_*i*_−μ_*j*_>0), was evaluated by the EAP (expected a posteriori) of the following generated quantity:


(1) $$u_{\mu_{i}-\mu_{j}>0}^{\left(t\right)}=u_{\mu_{i}>\mu_{j}}^{\left(t\right)}=\{\begin{array}{ll} 1 & \mu_{i}^{\left(t\right)}-\mu_{j}^{\left(t\right)}>0\\ 0 & \text{otherwises} \end{array}$$

## 3. Results

### 3.1. Perceived sweetness

**Figure 4:**
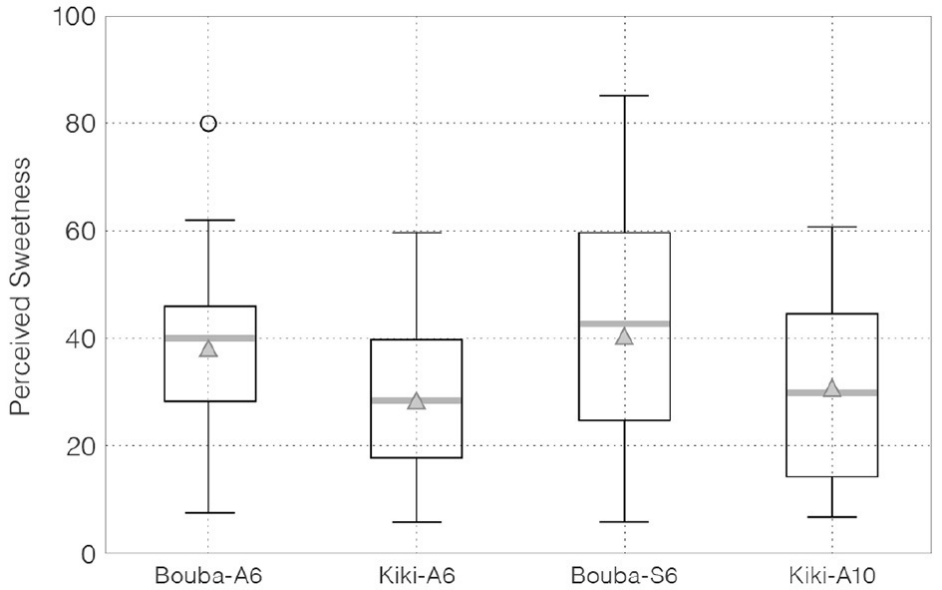
VAS (Visual Analog Scale) scores in participants' sweetness ratings. The boxes represent the middle 50% of the data, and the inside thick gray lines indicate the median value while the triangles indicate the mean value. The whiskers represent the maximum or minimum value of the data within 1.5 times the length of the box. Values not within this range were considered outliers (shown as individual data points).

Figure 4 shows the box-plot of participants' sweetness ratings for each edible sample.

Point estimates of the population mean of perceived sweetness using EAP for Bouba-A6, Kiki-A6, Bouba-S6, and Kiki-A10 were as follows: 38.52 (3.59) [31.21, 45.27], 28.90 (3.39) [21.96, 35.25], 40.63 (4.80) [31.58, 50.29], and 30.67 (3.73) [23.04, 37.76], respectively. Note that “( )” denotes the posterior standard deviation and “[ ]” denotes the 95% HDI (highest density interval).

**Figure 5:**
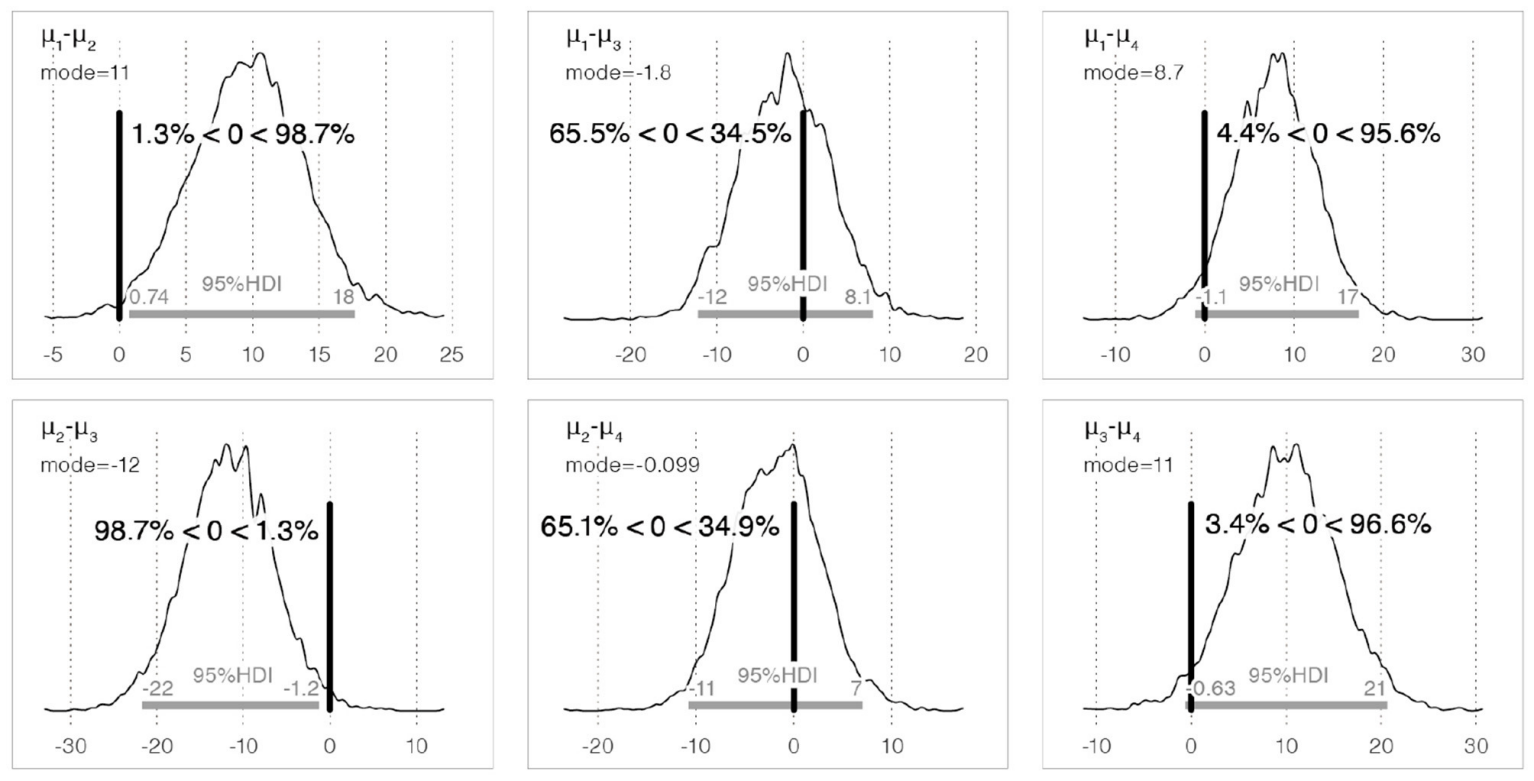
The posterior distribution of the difference in the population means for perceived sweetness between each sample, and the 95% HDI. Here, μ_*i*_ denotes the population mean of each edible sample (μ_1_: Bouba-A6, μ_2_: Kiki-A6, μ_3_: Bouba-S6, μ_4_: Kiki-A10). The vertical thick bar indicates the position of 0 and means the reference value. Note that *a%* < 0 < *b%* of μ_*i*_−μ_*j*_ next to the vertical thick bar denotes that the research hypothesis μ_*i*_>μ_*j*_ holds the probability at *b%* and μ_*i*_ < μ_*j*_ holds at *a%*.

Table 1 lists the probability that the sample in row *i* was perceived as sweeter than that in column *j*. Comparison of the population mean of perceived sweetness between the basic Bouba-shaped sample (Bouba-A6) and Kiki-shaped sample (Kiki-A6) (*H*_1_: Bouba-A6 > Kiki-A6) shows the Bouba-A6 was perceived as sweeter than the Kiki-A6 with a probability of 98.7%.^6^ Regarding *H*_4_ (Bouba-S6 > Bouba-A6), the probability of differences in the perceived sweetness due to minor shape differences was 65.5%.^7^ The posterior distribution of the difference in population means between each sample and the 95% HDI is shown in Figure 5.

**Table 1:** Probability that the sample in row *i* was perceived as sweeter than the sample in column *j*.

	**Sample**			
**Sample**	**Bouba-A6 (**μ_1_**)**	**Kiki-A6 (**μ_2_**)**	**Bouba-S6 (**μ_3_**)**	**Kiki-A10 (**μ_4_**)**
Bouba-A6 (μ_1_)	0	0.987	0.345	0.956
Kiki-A6 (μ_2_)	0.013	0	0.013	0.349
Bouba-S6 (μ_3_)	0.655	0.987	0	0.966
Kiki-A10 (μ_4_)	0.044	0.651	0.034	0

#### 3.1.1. Perceived sourness

**Figure 6:**
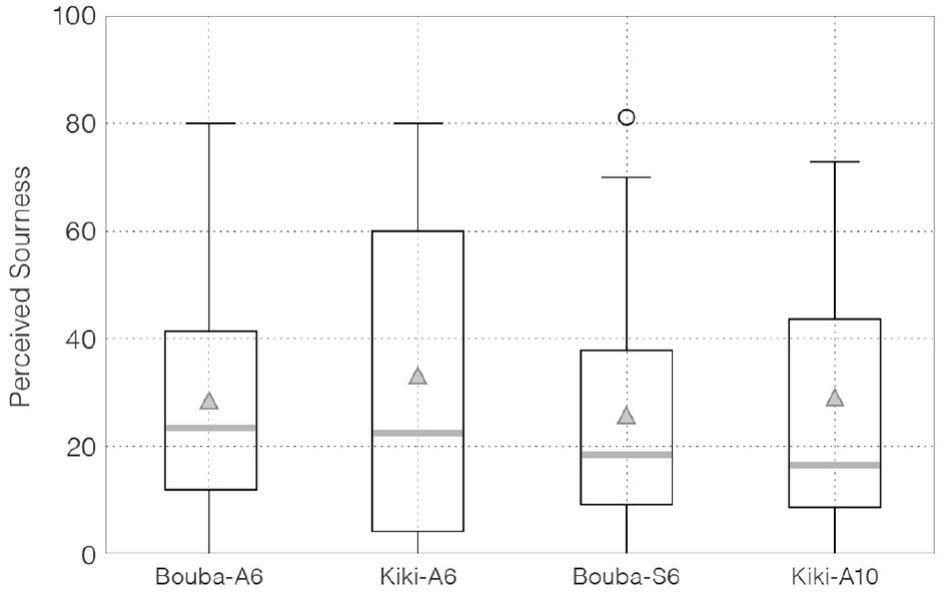
VAS scores in participants' sourness ratings.

Figure 6 shows a box-plot of participants' ratings of sourness for each edible sample.

Point estimation of population mean on perceived sourness by EAP regarding Bouba-A6, Kiki-A6, Bouba-S6, and Kiki-A10 are as follows: 28.49 (4.54) [19.20, 37.75], 33.45 (5.87) [22.41, 45.23], 25.75 (5.02) [15.88, 35.52], and 28.91 (5.40) [17.81, 39.36], respectively.

**Figure 7:**
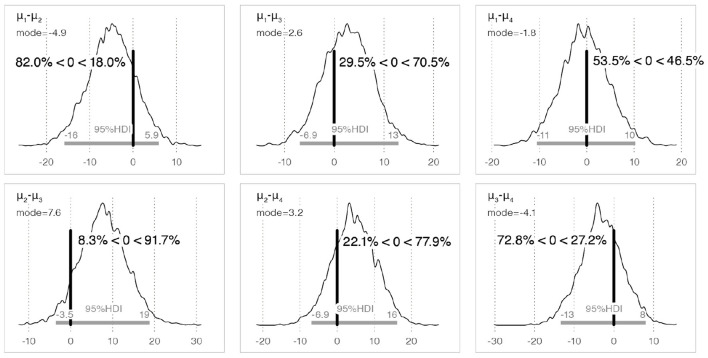
The posterior distribution of the difference in the population means for perceived sourness between each sample, and the 95% HDI.

Table 2 lists the probability that the sample in row *i* is perceived as more sour than that in column *j*. A comparison of the population mean for perceived sourness between the basic Kiki-shaped sample (Kiki-A6) and Bouba-shaped sample (Bouba-A6) (*H*_2_: Kiki-A6 > Bouba-A6) indicates that Kiki-A6 was perceived as more sour than Bouba-A6 with a probability of 82.0%.^8^ Regarding *H*_5_ (Kiki-A10 > Kiki-A6), the probability of differences in perceived sourness due to minor shape differences was 22.1%.^9^ The posterior distribution of the differences in the population means of each sample and the 95% HDI are shown in Figure 7.

**Table 2:** Probability that the sample in row *i* was perceived as more sour than the sample in column *j*.

	**Sample**			
**Sample**	**Bouba-A6 (**μ_1_**)**	**Kiki-A6 (**μ_2_**)**	**Bouba-S6 (**μ_3_**)**	**Kiki-A10 (**μ_4_**)**
Bouba-A6 (μ_1_)	0	0.180	0.705	0.465
Kiki-A6 (μ_2_)	0.820	0	0.917	0.779
Bouba-S6 (μ_3_)	0.295	0.083	0	0.272
Kiki-A10 (μ_4_)	0.535	0.221	0.728	0

### 3.2. Perceived bitterness

**Figure 8:**
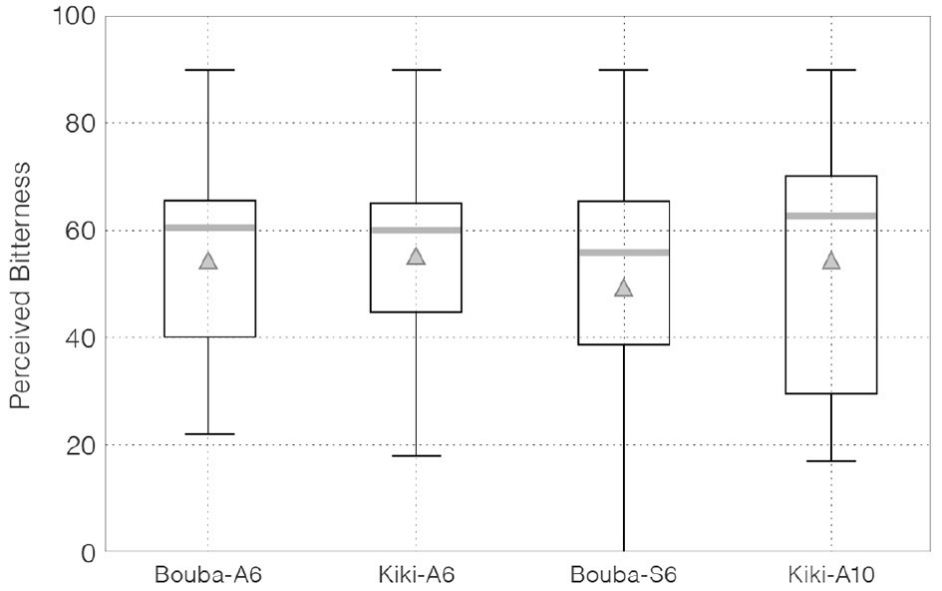
VAS scores in participants' bitterness ratings.

Figure 8 shows a box-plot of participants' ratings of bitterness for each edible sample.

Point estimates of population mean for perceived bitterness by EAP for Bouba-A6, Kiki-A6, Bouba-S6, and Kiki-A10 were as follows: 54.29 (3.84) [46.62, 61.74], 55.21 (4.06) [47.07, 62.94], 49.14 (5.11) [38.94, 58.90], and 54.40 (5.24) [44.39, 65.04], respectively.

Table 3 lists the probabilities that the sample in row *i* was perceived as more bitter than that in column *j*. Comparison of the population mean of perceived bitterness between the basic Kiki-shaped sample (Kiki-A6) and Bouba-shaped sample (Bouba-A6) (*H*_3_: Kiki-A6 > Bouba-A6) showed that Kiki-A6 was perceived as more bitter than Bouba-A6 with a probability of 57.3%.^10^ Regarding *H*_6_ (Kiki-A10 > Kiki-A6), the probability of differences in perceived bitterness owing to minor differences in shape was 43.7%.^11^

**Table 3:** Probability that the sample in row *i* was perceived as more bitter than the sample in column *j*.

	**Sample**			
**Sample**	**Bouba-A6 (**μ_1_**)**	**Kiki-A6 (**μ_2_**)**	**Bouba-S6 (**μ_3_**)**	**Kiki-A10 (**μ_4_**)**
Bouba-A6 (μ_1_)	0	0.427	0.822	0.494
Kiki-A6 (μ_2_)	0.573	0	0.851	0.563
Bouba-S6 (μ_3_)	0.178	0.149	0	0.206
Kiki-A10 (μ_4_)	0.506	0.437	0.794	0

**Figure 9:**
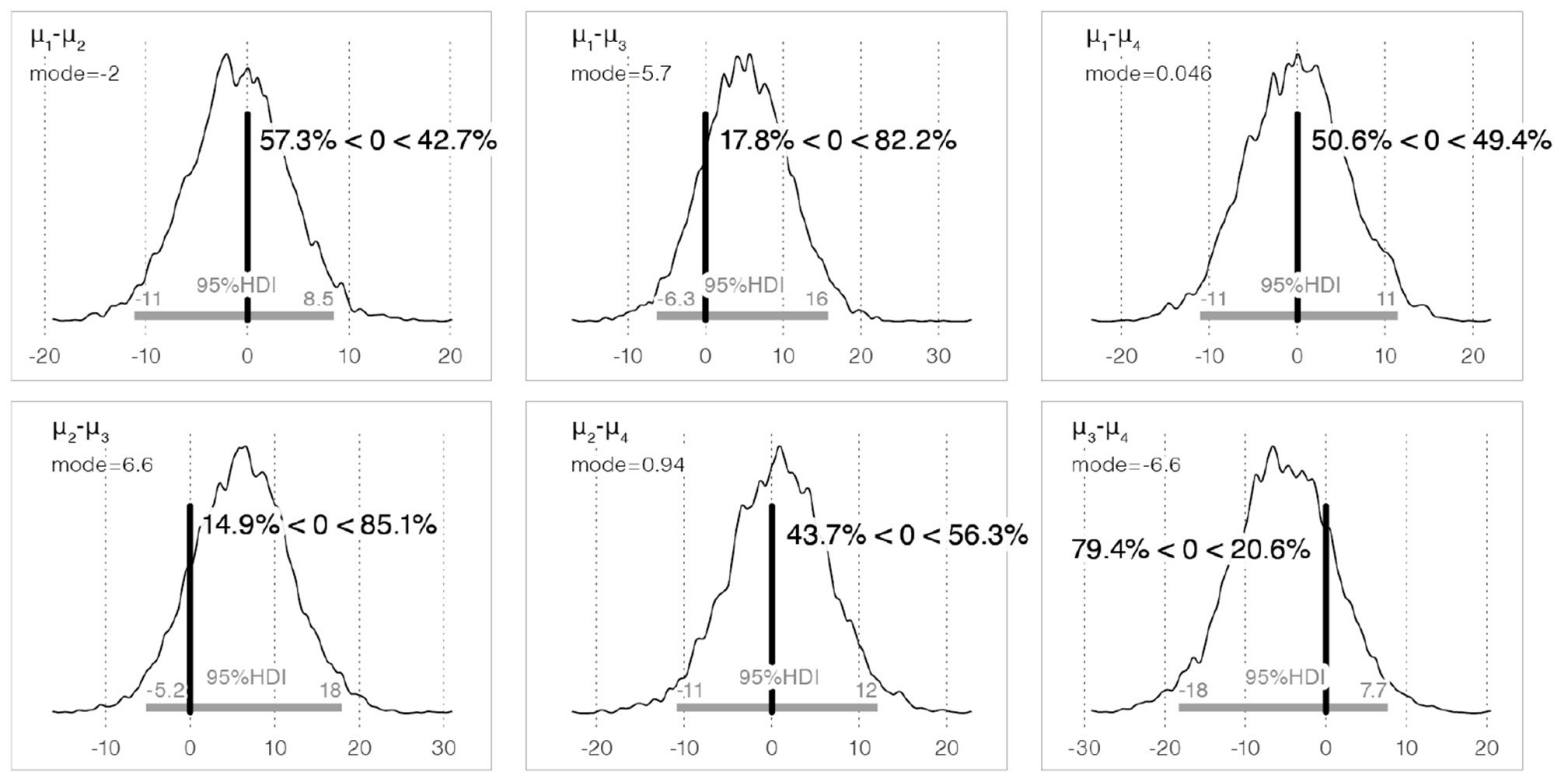
The posterior distribution of the difference in population means for perceived bitterness between each sample, and the 95% HDI.

The posterior distribution of the difference in population means between each sample and the 95% HDI is shown in Figure 9.

## 4. Discussion

### 4.1. Reflections on study results

This study investigated crossmodal correspondences between food shape and taste. We found that Bouba-shaped (round) chocolates were perceived as sweeter than Kiki-shaped (angular) chocolates. The results supported the first hypothesis (*H*_1_) and did not support the other hypotheses.

Regarding sweetness, our results supported *H*_1_ and did not support *H*_4_. The result for *H*_1_ is consistent with previous studies on crossmodal correspondences between shape and taste perception, in other words, basic Bouba-shaped (rounded) stimuli are perceived as sweeter than Kiki-shaped (angular) stimuli. In particular, our results are consistent with previous studies showing crossmodal correspondences between round shape and sweetness both in visual food perception (Deroy and Valentin, 2011; Ngo et al., 2011; Salgado-Montejo et al., 2015) and in actual consumption experiments (Lenfant et al., 2013; Cornelio et al., 2022). However, it is possible that the participants in our experiment did not perceive a sufficient difference in the tactile stimulation provided by the different edible samples, even though we designed the samples considering the tip distances at the shape extremities.

Regarding sourness, the results did not support *H*_2_ and *H*_5_. A closer look at the ratings of sourness (Figure 6) showed that the average values of each sample were lower than the other two tastes. Based on these results, the following hypothesis is proposed: it was unfamiliar or unclear for participants to rate the sourness of the chocolate samples, unlike ratings of sweetness and bitterness. This will play a role in constructing a further studies to understand correspondences between shape and sourness: one might use a standard sample as a sour taste reference, with participants rating the difference between each sample and this reference, so that participants can more easily estimate the range of differences.

Finally, regarding bitterness, the results did not support *H*_3_ and *H*_6_. Bitterness is the most complex of the basic tastes (sweet, sour, bitter, salty and umami) (Trivedi, 2012) and the ability to detect bitter tastes decreases with age (Drewnowski, 2001). Thus, it is possible that the age range of the participants in our study caused the lack of shape effects on bitterness.

To further explore the relationship between shape and taste, the four types of chocolate used in the experiment were analyzed separately for Bouba- and Kiki-shaped samples. Assuming (μ_*i*_, μ_*j*_)>(μ_*k*_, μ_*l*_), in which there is no up-down relationship between i and j or between k and l, but there is an up-down relationship between the two groups, the probability of this hypothesis being true can be obtained by calculating the EAP of the generation of the following Equation 2.


(2) $$\begin{array}{l} u_{\mu_{i}>\mu_{k}}^{\left(t\right)}\times u_{\mu_{i}>\mu_{l}}^{\left(t\right)}\times u_{\mu_{j}>\mu_{k}}^{\left(t\right)}\times u_{\mu_{j}>\mu_{l}}^{\left(t\right)} \end{array}$$

The overall probability of Bouba-shaped samples being perceived as sweeter than the Kiki-shaped samples, regardless of shape differences, was 90.0% (0.900 = 0.956 × 0.987 × 0.987 × 0.966). For some of those sample pairs, the 95% HDI included zero; however, the probability mass of the posterior distribution was concentrated in the positive or negative range. Therefore, a small effect might have been present. Regarding sourness and bitterness, the results showed no significant difference between the taste of Bouba-shaped and Kiki-shaped samples. The overall probability of Kiki-shaped samples being perceived as more sour than the Bouba-shaped samples, regardless of shape differences, was 29.2% (0.292 = 0.820 × 0.917 × 0.535 × 0.728). The overall probability of Kiki-shaped samples being perceived as more bitter than the Bouba-shaped samples, regardless of shape differences, was 19.6% (0.196 = 0.573 × 0.851 × 0.506 × 0.794).

We should note that generalizing the above results, namely the overall probability regardless of shape differences, may be challenging due to the limited samples. This limitation stems from our adoption of an asymmetric experimental design with a reduced sample size, which was intended to focus on examining the effects of the most likely effective parameters. Further study would be needed to test the influence of shape type, (a)symmetry, and a number of elements. In addition, we are still uncertain how much the change in taste/flavor itself affects generalization. In future work, we need to conduct experiments to see if the same round shape but different taste/flavor chocolate samples (e.g., milk chocolate, white chocolate) are perceived as sweeter than the angular shape.

### 4.2. Consideration for actual eating experiments

Although we have demonstrated the possibility of crossmodal correspondences between shape and taste, some limitations such as visual stimuli, food structure, individual differences, and expansion to other modalities and taste characteristics remain uncertain in our study. We briefly discuss them as below.

#### 4.2.1. Visual influence and tactile influence

One limitation of our study is that we could not distinguish between the effects of visual and tactile (mouthfeel) sensations of food shape on taste perception. It is known that visually perceived shape influences expectations regarding food taste. For example, consumers make judgments of food taste based on its packaging (Schifferstein, 2001). It is also known that haptic information through a hand plays a significant role in modulating food perception (Barnett-Cowan, 2010; Biggs et al., 2016). We focused on the effect of food shape on taste perception, and conducted an experiment in which participants ate four chocolate pieces with difference shapes. However, in the experiment, the participants could see the samples before eating to ensure that they were putting something edible into their mouths. Thus, we did not investigate the independent effects of visually perceived shape and tactile perceived shape. Further experiments are required to compare the results with and without observing stimuli. This would contribute to investigating crossmodal effects on taste, focusing only on shape stimuli perceived through the sense of touch.

Besides, we can also present a standard sample shape to clarify a taste evaluation baseline. Such an experimental design will help distinguish whether our results were perceived through food consumption or attributed through crossmodal correspondence, which our study did not clarify.

#### 4.2.2. Food structure, fragility, and amount

In our experiment, we used two main characteristic shapes (round and angular) to measure the influence of food shape. The weights of the edible samples were balanced, and the influence of differences in the amount of material was controlled. However, structural engineering properties such as brittleness, fragility, and elasticity of the edible samples, were not included in the design parameters. In their open-ended responses, several participants stated that the Kiki-shaped (angular) samples crumbled more easily and quickly transformed into small particles that melted more quickly. There is a possibility that the Kiki-shaped samples had areas of greater structural stress and quickly crumbled into smaller particles, even though the edible samples were not bitten.

On the other hand, a previous study found that the ease of crumbling depended on the direction in which the mechanical metamaterial chocolate was bitten, and that there was a positive correlation with the number of cracks and sensory ratings (e.g., how crunchy and easy to bite) of the overall taste (Souto et al., 2022). Another study showed that food with a honeycomb infill pattern requires longer to chew than food with a rectilinear pattern (Lin et al., 2020). A longer chewing time indicates that the food is structurally stronger. If this structure is related to the ease of melting, it could be utilized when studying how taste perception may be related to changes in structure.

Finally, we believe the ring-shaped samples used in our study could be generalized for comparing other shapes, such as circles vs. triangles. However, we assume that if the surface area expanded, the difference in taste perception would be smaller. As we pointed out in the Introduction and Section 2.1.1, we assumed that the filled samples had too much chocolate to be sufficiently discriminable in a previous study (Wang et al., 2017). Although we recommend using ring-shaped samples, the optimal amount of chocolate needs more exploration to be addressed in future research.

#### 4.2.3. Relationships between shapes and other taste characteristics

In this study, we focused on sweetness, bitterness, and sourness, which are basic human taste characteristics and tastes/flavors that chocolate typically has. However, there are other taste/flavor expressions such as spicy, oily, bland, astringency, and so on. Especially, astringency is a term that implies a characteristic of chocolate, wine, coffee, and so forth. When it comes to taste characteristics associated with chocolate, it may be possible to influence astringency by controlling the shape itself. Di Stefano and Spence (2022) noted that tactile stimuli produced by physical/mechanical contact between the food and the palate contribute to texture determination. These tactile stimuli include hardness, viscosity, elasticity and roughness. The study also discussed the relationship between roughness and astringency. In addition, Breen et al. (2019) showed that participants in the higher roughness sensitivity group responded to more subtle differences in chocolate roughness than those in the lower sensitivity group, suggesting that texture perception may be controllable. Roughness (which is angular), which tends to be perceived as unpleasant, like auditory and some visual and tactile stimuli, affects sensory and aesthetic responses in taste. Although our study highlighted results related to roundness and sweetness, it may be a good topic for future work to examine the possibility of manipulating the astringency evaluation by considering angularity as roughness.

Roughness, synonymous with angularity in this context, tends to be perceived as unpleasant and affects sensory and aesthetic responses to taste. Juravle et al. (2022) found that angular shapes, such as the Platonic solids, were typically associated with sour and bitter tastes. On the other hand, the study revealed a preference for sweetness and umami tastes with rounded shapes, such as spheres. This result suggests that the perception of different taste characteristics, including astringency, could be manipulated through the shape's angularity or roundness. Although our study primarily highlighted results related to roundness and sweetness, future work could consider the possibility of influencing astringency evaluations by manipulating the shape's roughness or angularity.

Moreover, regarding melting, Szubielska (2019) showed that chocolate was rated as tastier and more melt-in-the-mouth when eaten barefoot on a soft and smooth surface than a rough and hard surface. This study showed that taste perception also depends on the tactile properties of a contextual stimulus that occurs in the eating situation but is not related to the food itself, and this is in contrast to our finding that rough stimuli of the food itself influence melt-in-the-mouth. It will be interesting in future work to test which variable affects taste perception more between tactile contextual indicators or the tactile stimuli of the food itself.

#### 4.2.4. Individual and cultural differences

The other consideration is individual differences in taste perception. Individual differences, e.g., gender/age/sweetness preference, could influence taste perception, which Bertelsen et al. (2020) demonstrated through their large-scale experiment. The ratings of chocolate samples might have changed slightly during the experiment depending on whether one prefers sweet or bitter foods. The chocolate used in our experiment was 74% cacao, which is relatively bitter. Those who prefer sweeter chocolate may have perceived a stronger bitter taste when they tasted the edible samples. One of the participants stated, “*I found the sourness was not my thing because I like sweet chocolate*”, in the open-ended responses. In future work, we would like to investigate a correlation between individual differences and their evaluation of taste.

Geographical and cultural diversity should also be considered. A study focusing on the shape-taste matching of Bouba-Kiki showed that the effect size of each factor affecting shape-taste matching differed between the experimental participants from two different countries, namely the United Kingdom and Colombia (Salgado-Montejo et al., 2015), Taiwan and North America (Chen et al., 2016). Their study showed that shape-taste matching was similar across countries. On the other hand, Bremner et al. (2013) showed that the Himba of northern Namibia tended to associate less bitter chocolate samples with angular rather than rounded shapes, which is the opposite mapping to that shown by Westerners. It is prudent to continue to consider the possibility of cross-cultural differences in taste preferences (Prescott and Bell, 1995).

#### 4.2.5. Crossmodal harmony

Besides, there is a recent study that argues about the “harmony” of multiple modalities. Various food scientists have used the term “crossmodal harmony” to represent this notion. Some of them work on the combination of fragrances and flavors and use it to “*refer to fused, or united, percepts while at the same time talking of the balanced composition of a mixture of elements*” in a dish (Spence and Di Stefano, 2022). A congruent crossmodal will be associated with an affectively more positive value. As we obtained from the open-ended responses, the participants stated, “*I was curious to see unusual star-shaped chocolates*.” or “*This is the first time I had ever eaten chocolate in such a unique shape, and it is interesting*.” They enjoyed the opportunity to eat unique-shaped chocolate, which might positively affect them. It would be highly insightful as future work to comprehensively capture variables such as visual appearance, taste, tactile, and shape to consider evaluating their balance and perceptions. Such a comprehensive perspective regarding harmony will be meaningful for studying taste perception through actual food consumption.

## 5. Conclusion

This study focused on the crossmodal correspondence between the food shape and taste perception. Accordingly, in an empirical experiment, participants ate Bouba- and Kiki-shaped chocolate pieces that were created using a 3D food printer and sensory evaluations were performed. A Bayesian analysis of the survey results revealed that participants perceived the Bouba-shaped (round) chocolate pieces as sweeter than the Kiki-shaped (angular) pieces. Our findings follow previous research on the crossmodal correspondences that occur between visual stimuli and taste perception. The results of this study will contribute to broadening the scope of food shape design and the proposal of new dining methods and experiences, as well as developing further crossmodal research.

Our results may enrich the dining experiences in the metaverse, which is a line of research that has gained momentum in recent years (Jaller et al., 2021; Cornelio et al., 2022). Research exploring dining experiences in VR spaces has already begun and is expected to become an area that will increasingly attract in the future. Similarly, some researchers are exploring the association between 3D shapes and expected taste, liking, and so on (Chen et al., 2019), which could lead directly to the dining experiences in the metaverse. The results of the present study suggest that taste perception can be controlled by changing the shape of food. In addition to visually manipulating taste (Narumi et al., 2011; Narumi, 2016), our study may contribute to approaches that use non-digital elements (Harley et al., 2018) and augment the visual experience with other sensory stimuli (Velasco et al., 2018).

In addition, we believe that it is worth exploring the effects of such a multi-sensory tasting to enrich the dining experiences and contribute to providing sustainable food experiences (Pedersen et al., 2021) in a way that reveals not only what to eat but also how to eat. In fact, chocolate has the highest carbon footprint of any plant-based food, emitting 19 kg of CO_2_ equivalents per kilogram, compared to 17 kg for coffee and 8 kg for palm oil.^12^ Given that the shape of chocolate can affect the perception of sweetness, it may be possible to deliver the same or much more sweetness with a smaller amount of chocolate by changing its shape, as demonstrated by the ring-shaped pieces used in this study. From the perspective of 3D printing, or additive manufacturing, this production process has potential environmental benefits (Jambrak et al., 2021). It is possible to decentralize the production site, to produce the designed chocolate locally at the desired time/unit, and to reduce CO_2_ emissions during the production process, especially during transportation. If we can enjoy eating experiences with less chocolate, this will lead to a reduction in the ingredients needed and greenhouse gases from land use change and farm stage of the supply chain, which are the main contributors to emissions.

## Data availability statement

The original contributions presented in the study are included in the article/supplementary material, further inquiries can be directed to the corresponding author.

## Ethics statement

The studies involving human participants were reviewed and approved by OMRON Corporation Technology and Intellectual Property H.Q. Research Ethics Review Committee. The participants provided their written informed consent to participate in this study.

## Author contributions

KO, RG, AH, YU, and SY designed the study. KO, RG, and SY performed the experiment. KO designed and prepared the experimental materials with RG. RG analyzed the data with SY. KO and RG wrote the first draft of the manuscript under the supervision of SY with further review by AH and YU. All authors contributed to manuscript revision, read, and approved the submitted version.

## Footnotes

^1^ https://www.rhino3d.com

^2^ https://www.grasshopper3d.com

^3^ https://www.3dfoodbot.com

^4^The code is available here: https://github.com/omron-sinicx/ComputationalMouthfeel.

^5^ https://www.pymc.io/welcome.html

^6^For reference, the results of a *t*-test instead of a Bayesian analysis are presented here. Cohen's d is used to represent the effect size. The *t*-test results, *t*_(23)_ = 2.22, *p* = 0.018, *d* = 0.45, also show a significant difference between the two samples, supporting hypothesis *H*_1_.

^7^The *t*-test results, *t*_(23)_ = 0.47, *p* = 0.321, *d* = 0.10, suggest that the *p*-value is greater than the conventional significance level of 0.05. This value indicates a failure to reject the null hypothesis, as it does not provide significant statistical evidence to support the alternative hypothesis *H*_4_.

^8^The *t*-test results, *t*_(23)_ = 1.21, *p* = 0.120, *d* = 0.25, indicate that the *p*-value is greater than the conventional significance level of 0.05. The results do not provide substantial statistical evidence to support the alternative hypothesis *H*_2_.

^9^The *t*-test results, *t*_(23)_ = −0.99, *p* = 0.834, *d* = −0.20, indicate that the *p*-value is considerably higher than the conventional significance level of 0.05. Consequently, the results do not provide substantial statistical evidence to support the alternative hypothesis *H*_5_.

^10^The *t*-test results, *t*_(23)_ = 0.22, *p* = 0.414, *d* = 0.05, indicate that the results do not provide substantial statistical evidence to support the alternative hypothesis *H*_3_.

^11^The *t*-test results, *t*_(23)_ = −0.17, *p* = 0.568, *d* = −0.04, indicate that the results do not provide substantial statistical evidence to support the alternative hypothesis *H*_6_.

^12^ https://ourworldindata.org/food-choice-vs-eating-local
